# New Perception of Mitochondrial Regulatory Pathway in Parkinsonism – Ubiquitin, PINK1, and Parkin

**DOI:** 10.3389/fneur.2014.00247

**Published:** 2014-11-24

**Authors:** Diana Angelika Olszewska, Conor Fearon, Tim Lynch

**Affiliations:** ^1^Department of Neurology at the Dublin Neurological Institute, Mater Misericordiae University Hospital, Dublin, Ireland

**Keywords:** parkinsonism, ubiquitin, PINK1, parkin, phosphorylation

Mutations in PARK2, a gene encoding cytosolic E3 ubiquitin ligase parkin cause autosomal recessive Parkinsonism similar to mutations in the less prevalent PINK1 (PTEN induced putative kinase 1). Parkin and PINK 1 (Ser/Thr kinase) eliminate damaged mitochondria through mitophagy and mutations cause accumulation of impaired mitochondria and reactive oxygen species (1). PINK1, when activated by depolarization of the mitochondrial membrane potential, leads to phosphorylation and activation of E3 parkin ligase [Okatsu (2)]. PINK1 acts upstream to parkin accelerating latent E3 parkin activity and increasing accumulation of parkin on depolarized mitochondria in *Drosophila*. Presumably, PINK1-dependent phosphorylation of parkin at Ser65 accelerates E3 parkin activity. However, the substrate for PINK 1 phosphorylation of parkin has remained elusive.

Koyano et al. (1) demonstrated that PINK1 phosphorylates ubiquitin, which subsequently activates parkin on damaged mitochondria. PINK1-dependent parkin activation proceeds in two phases: phosphorylation of ubiquitin-like (UBL) domain of parkin at Ser 65, followed by phosphorylation at Ser 65 of ubiquitin itself. While UBL domain is known for keeping parkin inactivated, phosphorylation of Ser 65 activates parkin partially (3). Autoubiquitination is not promoted by non-phosphorylated ubiquitin. Kane et al. (4) and Kazlauskaite et al. (3) also confirmed phosphorylated ubiquitin as a parkin activator. These studies shed light on two further PINK1/parkin metabolism issues: (1) why does a phosphorylation-deficient mutation of parkin inhibit formation of a ubiquitin-ester (an intermediate product in the parkin activation pathway), (2) why does a phosphomimetic parkin mutant still require PINK1 for activation.

Koyano et al. used phosphate affinity (phos-tag) PAGE assay to identify a slower-migrating ubiquitin band, phosphorylated by PINK1 on damaged mitochondria (i.e., pre-treated a protonophore). Mass spectrometry analysis identified Ser 65 as the ubiquitin phosphorylation site. A yeast system was used to confirm that phosphorylated ubiquitin at Ser 65 is a parkin activator. Thus, ubiquitin acts not only as a substrate for phosphorylation but also activates parkin itself.

Koyano et al. proposed that parkin is fully activated by repression of the catalytic cysteine by RING0 domain unlocked by phosphorylated ubiquitin and UBL domain.

Parkinson’s disease is an incurable neurodegenerative condition. Defects in mitochondrial regulation have been implicated as one of the key elements in the etiology of parkinsonism. PINK 1 and parkin act as neuroprotective agents by maintaining mitochondrial homeostasis. Decreased antioxidant effect may be seen in other serious disorders, such as tumors where parkin expression is frequently diminished (5). Mechanism of interaction between these two proteins has been unclear. Koyano et al. used a yeast system to aid demonstration of the important role of phosphorylated ubiquitin acting as a long-searched for mediator between PINK1 and parkin.

The above results may lead to novel therapeutic options for Parkinson’s disease. Cornelissen et al. (6) proposed a treatment strategy based upon inhibition of ubiquitin-specific protease 15 (USP15), a deubiquitinating enzyme (DUB) counteracting Parkin-mediated autophagy, while Kazlauskaite et al. (3) suggested development of a parkin activator in the form of ubiquitin-like agent.

PINK1 driven phosphorylation of ubiquitin has important implications in understanding the underlying pathophysiological mechanisms of parkinsonism and to develop new treatment strategies for an incurable movement disorder.

## Conflict of Interest Statement

The authors declare that the research was conducted in the absence of any commercial or financial relationships that could be construed as a potential conflict of interest.
